# Design and Implementation of 24-GHz and 48-GHz VCOs Using Noise Filtering Technique in 90-nm CMOS

**DOI:** 10.3390/mi16060682

**Published:** 2025-06-05

**Authors:** Chen-Chih Ku, Sen Wang

**Affiliations:** Department of Electronic Engineering, National Taipei University of Technology, Taipei 10608, Taiwan; t111368053@ntut.org.tw

**Keywords:** CMOS 90-nm technology, voltage-controlled oscillators, push-push VCO, transmission line, slow-wave factor (SWF), noise filtering, reduce phase noise

## Abstract

This work proposes two voltage-controlled oscillators using noise-filtering technique. The first one is a 24-GHz voltage-controlled oscillator, and the second one is based on a push–push architecture with a λ/4 transmission line to further increase the frequency up to 48 GHz. The designs are implemented and verified in a standard 90-nm CMOS process. Typically, the current mirror transistor in the tail current has a nonlinear effect. When the transistor operates in the nonlinear region, noise will be introduced. Therefore, a set of LC filters with a resonant frequency at 2$f_{0}$ are added to the design of this section to filter the noise at 2$f_{0}$ through the capacitor to the ground. The measurement results show that the design of a single oscillator has an oscillation frequency of 24.37 GHz, a tuning range of 6.5%, and a phase noise of −97.19 dBc/Hz @1MHz. The measurement results of the push–push architecture show that the double oscillation frequency is 49.8 GHz, the tuning range is 7.2%, and the phase noise is −80.52 dBc/Hz @1MHz. The chip areas of 24-GHz LC VCO and 48-GHz push–push LC VCO are 0.68 mm × 0.69 mm and 0.7 mm × 0.7 mm, respectively.

## 1. Introduction

Voltage controlled oscillators (VCOs) play an important role in the design of microwave circuits. Its function is to provide a stable local oscillation (LO) signal. The VCOs are required to have low phase noise, low power consumption, wide tuning range and high output power from the design considerations. In radio frequency (RF) systems, low phase noise can improve the sensitivity and linearity of the receiver, which is crucial for some high-precision RF and digital signal processing systems. However, the tail current in the current mirror transistor has a nonlinear effect, which mixes the second harmonic signal with the fundamental signal to produce a signal with a frequency close to f_0_. This new signal will shift the 2f_0_ noise to near the oscillation frequency. In order to reduce phase noise, using LC noise filtering circuit can effectively reduce phase noise [1,2,3] and eliminate the harmonic noise between the current source and the CMOS cross-coupling [4,5,6]. In Section 2 of this paper, we used a set of LC filters with a resonant frequency of 2f0 to filter out the noise at 2f_0_, through the capacitor to the ground, and reduce the phase noise of the circuit [7].

However, the output signal of a single frequency oscillator is limited by the cut-off frequency of the transistor, which means that the output oscillator signal cannot reach a higher frequency band. Therefore, the push-push architecture used "λ"/4transmission line can improve the output signal limitation of the characteristics of the transistor itself [8], This paper extends the proposed VCO architecture, design a "λ"/4 transmission line combined with an LC voltage-controlled oscillator to implement a Push-Push voltage-controlled oscillator [9,10] and we also discuss how to use the slow wave factor (SWF) equation to design low-attenuation transmission lines [11]. The remainder of this paper is organized as follows. Section 2 describes the design and analysis of the two proposed VCOs. In Section 3, the implementation and measurements of the two VCOs detailed. Finally, Section 4 concludes this paper.

## 2. Design and Analysis

**Figure 1:**
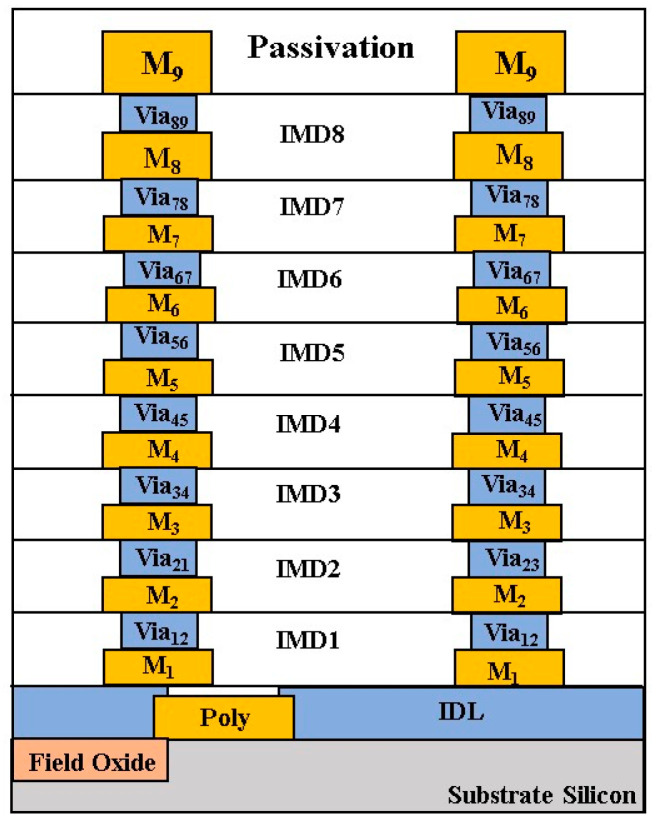
Cross-section of a standard 90-nm CMOS process.

A standard 90-nm CMOS process offers one poly layer and nine metal layers, as shown in Figure 1. The thicknesses of the top metal layers (M_9_) and (M_8_) are 0.85 μm, the thicknesses of the metal layers (M_2~7_) are all 0.31 μm, while the metal layer (M_1_) is 0.24 μm. Typically, the M_1_ layer is also known as the bottom metal layer. Each layer of metal can be connected by Via layers. The poly layer is for the transistor’s gate and thus above the substrate.

### 2.1. The Proposed 24-GHz VCO

**Figure 2:**
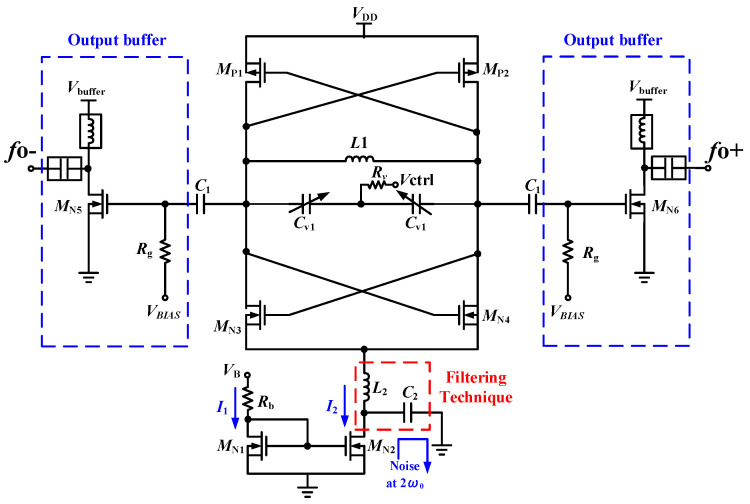
Schematic of the proposed CMOS 24 GHz VCO.

The schematic of 24 GHz VCO with low phase noise is shown in Figure 2. This circuit is a voltage-controlled oscillator composed of CMOS architecture, *M*_N3_, *M*_N4_ and *M*_p1_, *M*_p2_ are the cross-couple pair, and the LC resonant cavity is composed of *L*_1_ and *C*_v1_. The capacitance change of the variable capacitor in the circuit mainly determines the overall tuning range of the VCO. However, for the circuit to oscillate stably, the negative impedance of the cross-coupled pair needs to offset the positive impedance of the resonant cavity. Therefore, the negative impedance of this voltage-controlled oscillator is created by the cross-coupling of the NMOS and PMOS to the transistor.

The NMOS current mirror can use voltage to limit the size of the tail current, limiting the total power dissipated by the voltage-controlled oscillator. The channel width ratio of the transistors *M*_N1_ and *M*_N2_ is 16:1. However, the current mirror transistor in the tail current has a nonlinear effect. When the transistor operates in the nonlinear region, noise will be introduced. The second harmonic signal is mixed with the fundamental frequency signal, and the signals are multiplied to produce a signal whose frequency is close to the oscillation frequency, and this new signal will transfer the 2f_0_ noise to near the oscillation frequency, reducing the phase noise of the circuit [4]. Therefore, a set of LC filters with a resonant frequency at 2f_0_ are added to the design of this section to filter the noise at 2f_0_ through the capacitor to the ground. The inductor provides a high impedance of the circuit near the resonant frequency and blocks the passage of the 2*f*_0_ signal. The function of the capacitor is to compose a resonant circuit with the inductor [5]. Finally, open-drain buffers *M*_N5_ and *M*_N6_ are used at the output, and bias Tee is used externally, which can be used to isolate RF and DC signals and enhance the output power.

**Figure 3:**
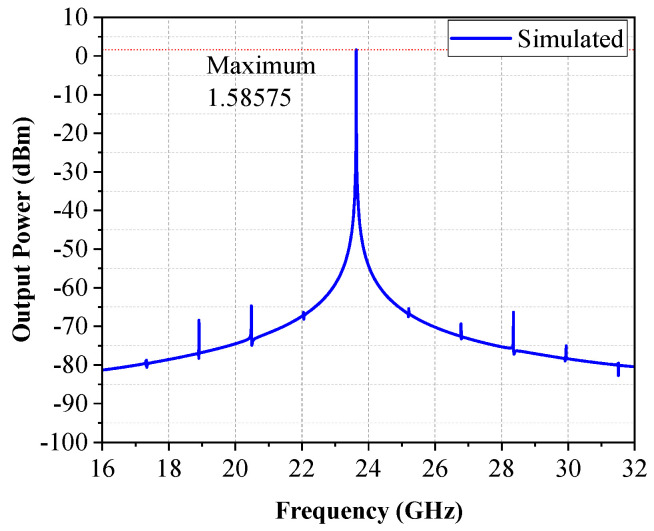
Simulated output spectrum.

**Figure 4:**
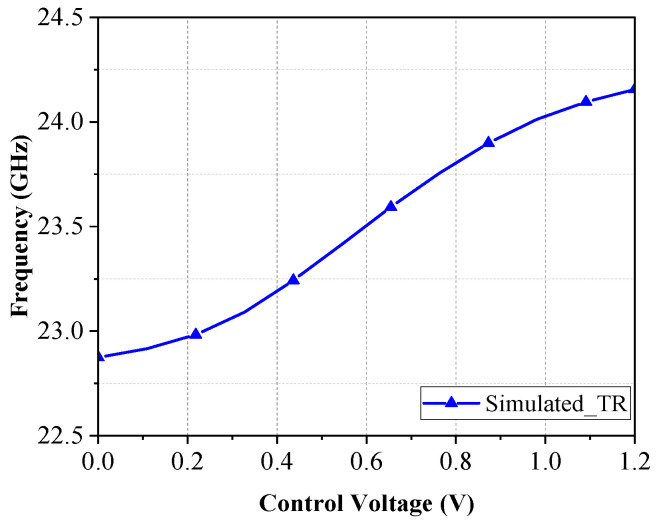
Simulation of VCO tuning range.

**Figure 5:**
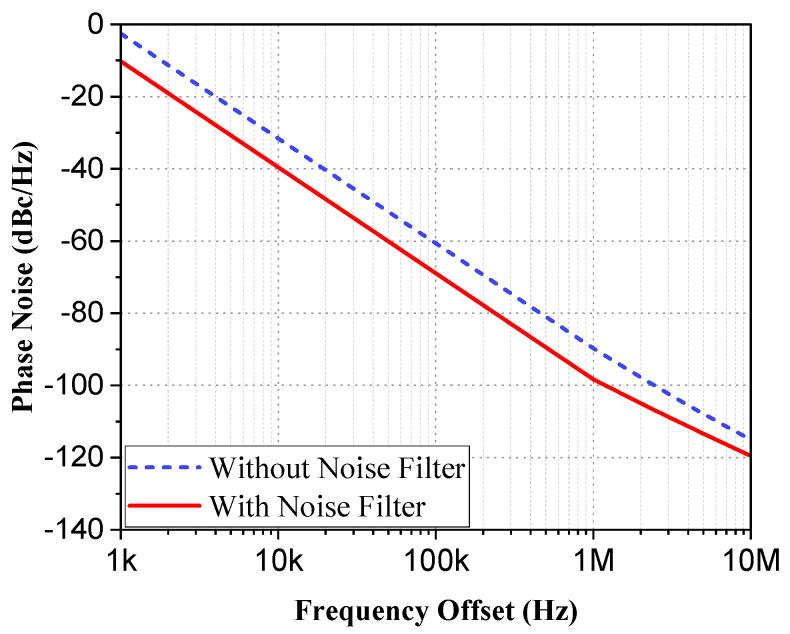
Phase noise comparison chart after adding noise filter circuit.

Figure 3 is the simulated output spectrum of the voltage-controlled oscillator after the implementation of the buffer amplifier. The output oscillation frequency is 23.63 GHz and the output power is 1.58 dBm when Vctrl is 0.6 V, while the frequency range is from 22.8~24.15 GHz, as shown in Figure 4, and the tuning range is 5.45%. Figure 5 shows the simulated results of phase noise after adding a noise filter circuit. The phase noise simulation result is −95.8 dBc/Hz at a carrier offset of 1 MHz. Obviously, the phase noises are improved by about 8.3 dB by adding the noise filter circuit.

### 2.2. The Proposed Push–Push 48-GHz VCO

#### 2.2.1. The λ

/4 Transmission Line

With the development of communication systems, low-loss and high slow-wave transmission lines are required for improving circuit performances and miniaturization. In this design, a λ/4 transmission line was combined with an LC voltage-controlled oscillator to implement a push–push VCO. Firstly, the characteristic impedance value of the transmission line must be calculated, as shown in Equation (1).

(1) $$Z_{C}=Z_{0}\sqrt{\frac{{\left(1+S_{11}\right)}^{2}-{S_{21}}^{2}}{{\left(1-S_{11}\right)}^{2}-{S_{21}}^{2}}}$$

Secondly, according to Equation (2), various electrical characteristics of the transmission line can be extracted. First, we need to extract the effective propagation constant γ of the transmission line, and the effective propagation constant is denoted by γ = $\alpha +j\beta_{g}$, where β is the phase constant, α is the attenuation constant, and L is the physical length of the transmission line.

(2) $$e^{\gamma L}=\frac{1-{S_{11}}^{2}+{S_{21}}^{2}\sqrt{{\left(1+{S_{11}}^{2}-{S_{21}}^{2}\right)}^{2}-{\left(2{\;S}_{11}\right)}^{2}}}{2{\;S}_{21}}$$

**Figure 6:**
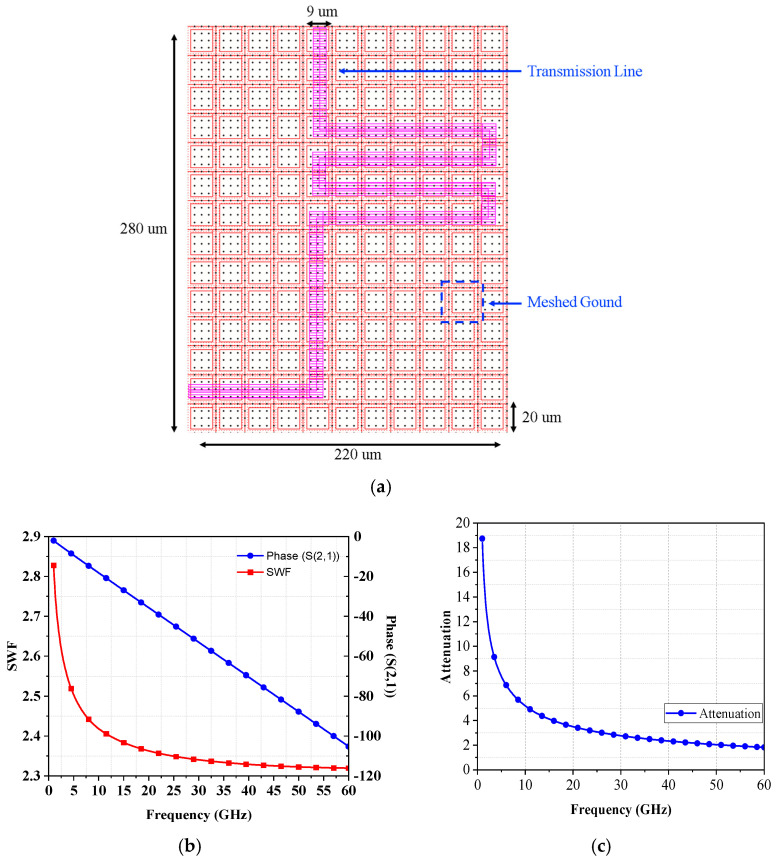
(**a**) The proposed λ/4 transmission line in CMOS 90-nm process, (**b**) slow-wave factor (SWF), and (**c**) attenuation results of 50-Ω λ/4 transmission line.

In the design of transmission lines, the slow wave factor (SWF) plays an important part [8]. The slow wave factor refers to the measurement of the loss and distortion experienced by electromagnetic waves propagating in transmission lines. We can calculate the length of the λ/4 transmission line at 48 GHz to be 672 um based on Equation (3), as shown in Figure 6a. The transmission line is winding according to this length. The slow wave factor and phase after the simulated winding are shown in Figure 6b, and the phase attenuation is shown in Figure 6c.

(3) $$\mathrm{SWF}=\frac{\beta_{g}}{\beta_{0}}=\frac{\lambda_{0}}{\lambda_{g}}=\sqrt{\varepsilon_{r}u_{r}}\approx \sqrt{\varepsilon_{r}}$$

#### 2.2.2. The Push–Push VCO

**Figure 7:**
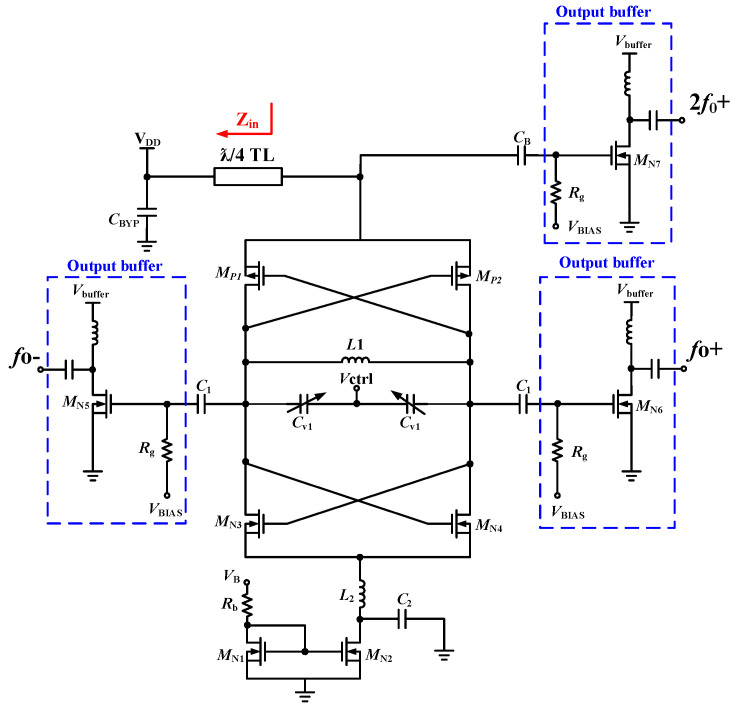
Schematic of the proposed 48 GHz Push-Push VCO.

The schematic of the 48-GHz push–push VCO is shown in Figure 7. This VCO is composed of the cross-couple pair of *M*_N3_, *M*_N4_, *M*_P1_, and *M*_P2_, the LC tank of *L*_1_ and *C*_v1_, the NMOS current mirror of transistors *M*_N1_ and *M*_N2_ with a channel width ratio of 16:1, and the LC noise-filtering circuit. In the traditional LC architecture, the inductor used as the resonant circuit can usually be replaced by a λ/2 transmission line. However, this transmission line is the state of one wavelength, so the oscillation frequency at 2*f*_0_ is in phase and multiplies, *f*_0_ is the differential signal cancellation, and an oscillation signal of 2*f*_0_ is generated; this is extracted through the AC coupling capacitor *C*_B_ through open-drain buffers *M*_N7_ to output a double-frequency oscillation signal, and λ/4 of the transmission line in the circuit is in the 2*f*_0_ state. It functions as an RF Choke in the circuit to isolate RF signals. Z_in_ represents the input impedance of the transmission line. The load impedance *Z*_L_ in Equation (4) is 0, *Z*_0_ is the characteristic impedance of the transmission line, and L is λ/4. It can be known that the signal under the λ/4 transmission line is infinite and is an open circuit state.

(4) $$Z_{\mathrm{in}}=Z_{0}\;\times \frac{Z_{L}+jZ_{0}\tan\beta L}{Z_{0}+jZ_{L}\tan\beta L}=jZ_{0}\tan\beta L=jZ_{0}\tan\frac{2\pi }{\lambda }\times \frac{\lambda }{4}\approx \infty$$

## 3. Implementation and Experimental Results

Both the two VCOs that the 24-GHz design adds a noise filter to reduce phase noise and the 48-GHz design that uses the push-push architecture are fabricated using the TSMC 90-nm CMOS process. The process provides a single poly layer and nine metal layers (1P9M) for interconnections. The chip size, including RF and DC testing pads of 24-GHz VCO, is 0.68 mm × 0.69 mm, and the push–push VCO is 0.7 mm × 0.7 mm, respectively.

**Figure 8:**
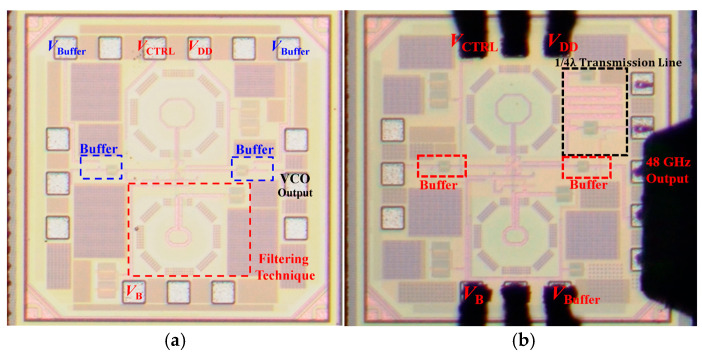
Chip photo of the proposed (**a**) 24-GHz and (**b**) 48-GHz push–push VCOs.

On-wafer measurements were performed to characterize the output spectrum, tuning range and phase noise. For the 24-GHz VCO measurements, two ground-signal-ground (GSG) RF probes were used for differential output at the power supply end, one PGPPGP DC probe for V_DD_, V_ctrl_ and for the two output buffer gate terminals’ DC supply, and one PGP probe DC probe for the current mirror bias V_B_. There are bypass capacitors on the DC lines to reduce low-frequency noise, as shown in Figure 8a. As for the 48-GH z push–push VCO, one GSGSG RF probe was used for the 48-GHz output signal, one PGP DC probe for V_DD_ and V_ctrl_, one PGP DC probe for output buffer gate terminals, and the current mirror bias V_B_, as shown in Figure 8b.

**Figure 9:**
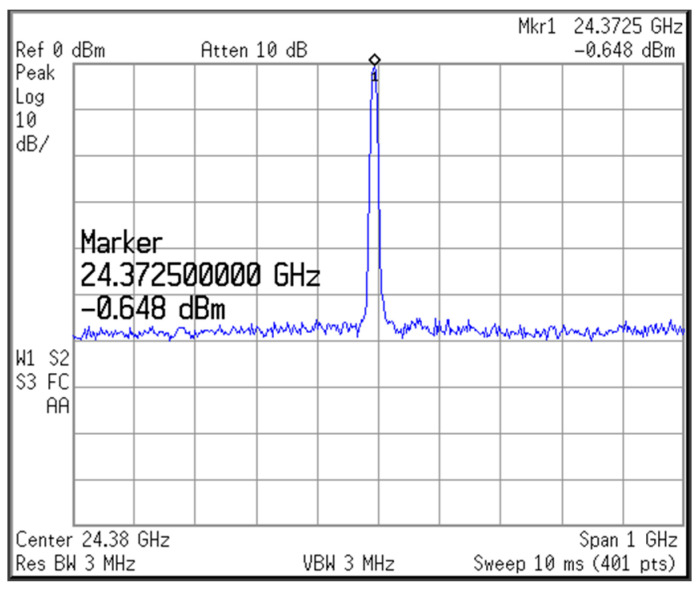
Measured output spectrum of the proposed 24 GHz VCO.

**Figure 10:**
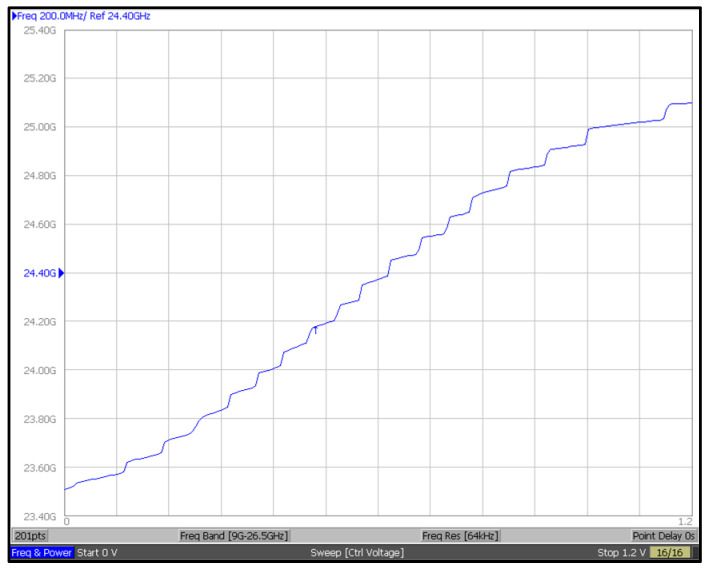
Measured tuning range of the proposed 24 GHz VCO.

**Figure 11:**
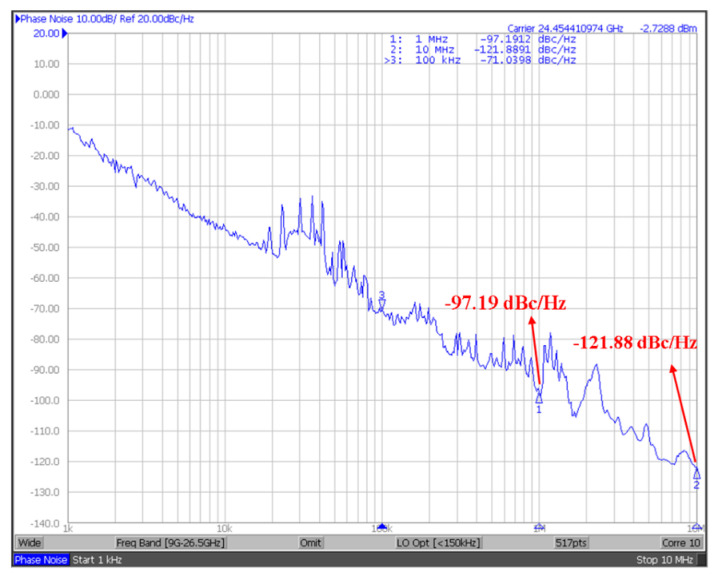
Measured phase noise of the proposed 24 GHz VCO.

Figure 9 shows the measured output spectrum of the proposed VCO; the measurement DC current of VCO without buffer is 5.1 mA, 6.12 mW. When V_ctrl_ is 0.6 V, the output frequency of the voltage-controlled oscillator is 24.37 GHz and the output power is −0.65 dBm. Figure 10 shows the measured tuning range of the proposed 24 GHz VCO. When the control voltage V_ctrl_ varies from 0 to 1.2 V and the frequency range is 23.51~25.09 GHz, the tuning range is 6.5%. When measuring the tuning range, we set the control voltage sweep points to 201 points. This will cause the VCO output frequency to change discontinuously when the sweep range is wide. If a higher number of points (such as 801 points) is used for scanning, the curve will become smoother and more continuous. Figure 11 shows the measured phase noise of the proposed 24 GHz VCO; the phase noise measured at a 1 MHz offset is −97.2 dBc/Hz, and that measured at a 10 MHz offset is −121.9 dBc/Hz.

**Figure 12:**
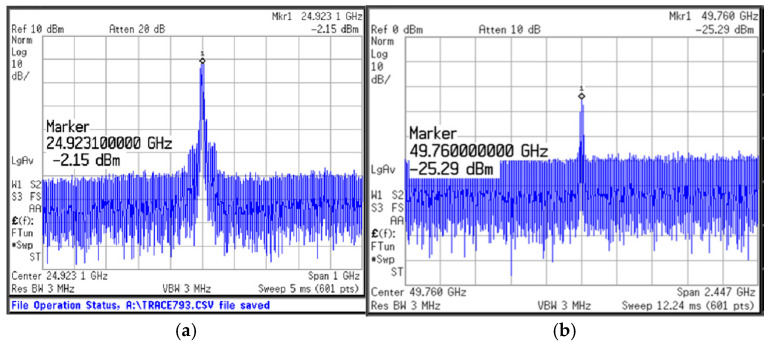
Measured output spectrum of the proposed push–push VCO: (**a**) *f*_0_ and (**b**) 2*f*_0_.

**Figure 13:**
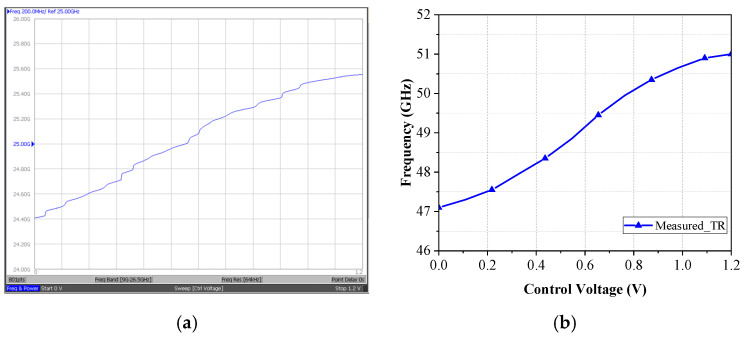
Measured tuning range of the proposed push–push VCO: (**a**) *f*_0_ and (**b**) 2*f*_0_.

**Figure 14:**
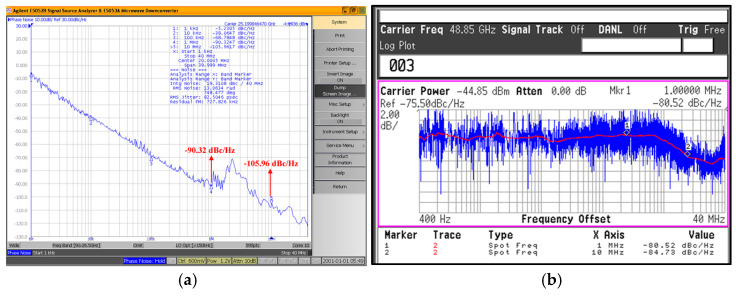
Measured phase noise of the proposed push–push VCO: (**a**) *f*_0_ and (**b**) 2*f*_0_.

Figure 12 shows the measured output spectrum of the proposed push–push VCO. The measurement DC current of the design without buffers is 5.74 mA, and *P*_out_ is 6.89 mW. In Figure 12a, when V_ctrl_ is 0.6 V, the output frequency of the *f*_0_ is 24.92 GHz and the output power is −2.15 dBm. In Figure 12b, when V_ctrl_ is 0.6 V, the output frequency of the 2*f*_0_ is 49.76 GHz and the output power is −25.29 dBm. Figure 13 shows that the measured tuning range of the push–push VCO. With the control voltage *V*_ctrl_ varying from 0 to 1.2 V, the frequency range of *f*_0_ is 24.1~25.9 GHz and that of 2*f*_0_ is 47.1~51 GHz. Figure 14 shows the measured phase noise of the VCO. In Figure 14a,b, the measured phase noises of *f*_0_ at a 1 MHz offset and of 2*f*_0_ at a 1 MHz offset are −90.32 dBc/Hz and −80.52 dBc/Hz, respectively.

In general, good agreements between the simulated and measured results can be observed. Table 1 summarizes previously reported CMOS VCOs. Among these works, the proposed 24-GHz design features good performances in the output power, power consumption, phase noise and FOM. The simulation and measurement results both show that the overall phase noise can be reduced after adding the noise-filtering circuit. The comparison table of the proposed push–push VCO is shown in Table 2. The proposed 48-GHz design has a high output power, low power consumption and acceptable phase noise performances. Equation (5) explains how the FOM in Table 1 and Table 2 is defined and calculated.

(5) $$\mathrm{FOM}=\mathrm{PN}-20\log\left(\frac{f_{\mathrm{OSC}}}{∆\;f}\right)+10\log\left(\frac{P_{\mathrm{VCO}}}{1\;\mathrm{mW}}\right)$$

## 4. Conclusions

Two low phase noise voltage-controlled oscillators were developed and implemented using CMOS 90-nm process. One is implemented in the 24-GHz frequency band, and the other is based on a push–push topology with a λ/4 transmission line that ranges up to the 48-GHz band. The good agreement between the simulated and measured results demonstrates the feasibility of the design concept. In both circuit architectures, adding the LC filtering technique at the tail current source can effectively reduce circuit phase noise; furthermore, in the push–push VCO, the use of the quarter-wavelength transmission line architecture can effectively reduce power consumption. This paper also proves that the push–push architecture can achieve a high-frequency oscillation in even a standard process, which can be applied to the RF transceiver system in the millimeter wave frequency band and to short-range radar applications to provide stable local signals.

## Figures and Tables

**Table 1:** Comparison of previously reported CMOS VCOs.

Ref.	[12]	[13]	[14]	This Work
**Technology (nm)**	90	65	130	90
**Supply (V)**	1.5	1.2	1.2	1.2
**Frequency (GHz)**	14.62–15.5	21.4–22.4	23.2–29.4	23.51–25.09
**Power Consumption (mW)**	9.13	16	36.5	6.12
**P_OUT_ (dBm)**	−4.12	N/A	−11	−0.65
**Tuning Range (%)**	5.8	11	26.5	6.5
**Phase Noise (dBc/1MHz)**	−94.86	−93.4	−92.6	−97.19
**FOM**	−168	−179.5	−165.4	−177.1
**Chip Core Area (mm^2^)**	N/A	0.64	0.12	0.47

**Table 2:** Comparison of previously reported push–push or millimeter-wave CMOS VCOs.

Ref.	[15]	[16]	[17]	This Work
**Technology (nm)**	90	90	180	90
**Supply (V)**	0.6	1.2	1.8	1.2
**Frequency (GHz)**	28.26/56.52	21/55	69.3	24.9/49.8
**Power Consumption (mW)**	7.08	14	27.5	6.89
**P_OUT_ (dBm)**	−13.74/−40.47	−30.5/−21.17	−31.46	−2.15/−25.29
**Tuning Range (%)**	8.83	N/A	5.2	7.2
**Phase Noise (dBc/1MHz)**	−95.62/−87.83	−100.8/−86.7	−75.23	−90.32/−80.52
**FOM**	−176.1/−174.3	−175.8/−170	−158.3	−169.9/−166.2
**Chip Core Area (mm^2^)**	0.42	0.009	0.208	0.49

## Data Availability

The original contributions presented in the study are included in the article, further inquiries can be directed to the corresponding author.
